# Colorimetric Fingerprints of Gold Nanorods for Discriminating Catecholamine Neurotransmitters in Urine Samples

**DOI:** 10.1038/s41598-017-08704-5

**Published:** 2017-08-15

**Authors:** Somayeh Jafarinejad, Mahmoud Ghazi-Khansari, Forough Ghasemi, Pezhman Sasanpour, M. Reza Hormozi-Nezhad

**Affiliations:** 1 Department of Medical Physics and Biomedical Engineering, Faculty of Medicine, Shahid Beheshti University of Medical Sciences, Tehran, Iran; 2 Department of Pharmacology, School of Medicine, Tehran University of Medical Sciences, P.O. Box, 13145-784 Tehran, Iran; 3 Department of Chemistry, Sharif University of Technology, Tehran, 11155-9516 Iran

## Abstract

Catecholamine neurotransmitters, generally including dopamine (DA), epinephrine (EP) and norepinephrine (NE) are known as substantial indicators of various neurological diseases. Simultaneous detection of these compounds and their metabolites is highly recommended in early clinical diagnosis. To this aim, in the present contribution, a high performance colorimetric sensor array has been proposed for the detection and discrimination of catecholamines based on their reducing ability to deposit silver on the surface of gold nanorods (AuNRs). The amassed silver nanoshell led to a blue shift in the longitudinal localized surface plasmon resonance (LSPR) peak of AuNRs, creating a unique pattern for each of the neurotransmitters. Hierarchical cluster analysis (HCA) and linear discriminate analysis (LDA) pattern recognition techniques were employed to identify DA, EP and NE. The proposed colorimetric array is able to differentiate among individual neurotransmitters as well as their mixtures, successfully. Finally, it was shown that the sensor array can identify these neurotransmitters in human urine samples.

## Introduction

Catecholamine neurotransmitters with a catechol structure (e.g., dopamine (DA), epinephrine(EP) and norepinephrine(NE)) play fundamental physiological roles in the central and peripheral nervous system^1, 2^. Monitoring neurotransmitters level is used for assessing the nervous system function and clarifying the disease mechanisms (if any). Moreover, the detection of this class of catecholamines is of high importance in early clinical diagnosis of various neurological diseases^3^ such as neuroblastomas^4^, pheochromocytomas^5^, Parkinsonism^6^, Schizophrenia^7^, Alzheimer^8^, Down’s syndrome and multiple sclerosis^9–11^.

However, due to their low concentration, easy oxidation and similar structures, it is usually difficult to find reliable and sensitive methods for simultaneous detection of catecholamine neurotransmitters. Until now, several analytical methods have been reported for the detection of neurotransmitters; for instance: electrochemical^12, 13^, enzyme-based^14, 15^, optical methods (e.g., fluorescent and colorimetric probes)^16–18^, mass spectrometry^19^ and chromatography (such as gas chromatography, liquid chromatography^20^ and high performance liquid chromatography)^3, 21, 22^.

Each of these strategies suffers from limitations such as inadequate selectivity, complicated instrumentation and time consuming sample preparations. In order to overcome these drawbacks and achieve a simple, reliable and fast method for simultaneous determination of catecholamine neurotransmitters, an idea is to utilize “array” based sensing platforms^23^. By mimicking the mammalian olfactory system, array based sensing methods exploit cross-reactive semi-selective sensing elements to provide a unique pattern for each analyte of interest^24^. Thus, unlike lock-and-key methods in which specific interactions are used, sensor arrays use nonspecific interaction profiles. This high-throughput approach has been employed widely for the recognition and determination of various analytes^24–27^. Multivariate analysis methods are used to handle the large amount of data produced by the array and for extracting fingerprint patterns of the analytes^28, 29^. Among different types of sensor arrays, colorimetric sensor arrays have attracted considerable attention owing to their sensitivity, simplicity, low cost and fast. a large variety of analytes^30^ including: pathogenic bacteria and fungi^31^, biomolecules^23, 32–34^, toxic materials^35^, different foods and beverages^36, 37^ have been discriminated using colorimetric sensor arrays, in which either conventional chromophores or nanoparticles have been used as recognition elements.

Plasmonic nanomaterials, due to their unique optical properties, have recently attracted intense attention as sensor elements. These nanostructures exhibit intensive localized surface plasmon resonance (LSPR) within the visible or near IR regions^38^ which can be finely tuned by changing the size, shape and composition of NPs in addition to their local/environmental dielectric constant^39, 40^. GNRs as powerful candidates in sensor array design reveal two LSPR peaks: longitudinal and transverse^41^. The former can be extremely altered in a broad spectral range from visible to near-IR, depending on the aspect ratio (AR) of GNRs^42, 43^. Increasing the AR results in a red shift, while a blue shift is observed when the AR is decreased^44–48^. Among different approaches for this purpose; one strategy is to deposit silver nanaparticles on the surface of GNRs. So far, this sensing mechanism has been applied in the detection of Escheichia coli^44^, enzyme activity^49^, perishable products^42^, and in immunoassays^50^. To the best of our knowledge, the use of this strategy within a sensor array design has not been reported yet.

In this study, a novel array based platform has been proposed for simultaneous colorimetric determination of catecholamine neurotransmitters. The presence of DA, EP and NA resulted in the accumulation of silver nanoparticles on top of GNRs. Depending on the concentration of neurotransmitters; different colors were observed resulting from different variations in the aspect ratios. Distinct patterns correlated to each neurotransmitter were collected by recording the absorbance spectra of GNRs at different concentration values. Color difference map and chemometric methods, including linear discrimination analysis (LDA), hierarchical cluster analysis (HCA) and principal component analysis (PCA) were employed for analyzing the array responses. The analytical performance of the developed array was further confirmed by testing complex mixtures and real urine samples.

## Results and Discussion

### Principle and fabrication of the sensor array

**Figure 1:**
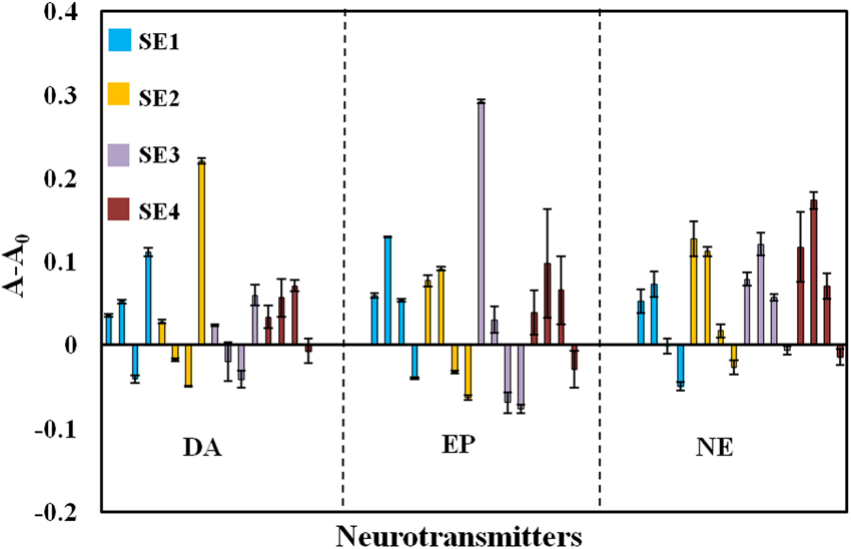
Unique response pattern bar plots for dopamine, epinephrine and norepinephrine at concentration of 20 µg mL^−1^ in four spectrum wavelengths (i.e., 520, 850, 950 and 1000 nm).

The growth of gold nanoparticles through the oxidation of catecholamine neurotransmitters, as active reducing agents, has been previously reported^18^. As a result of gold nanoparticle’s growth, the alteration of the absorption spectra as well as the color change in gold nanoparticle solution can be clearly observed. Herein, we have reported the use of catecholamine neurotransmitters, as active reducing agents, for the formation of silver nanoshell on the surface of gold nanorods. The new optical properties of the Au@Ag core-shell make it possible to quantify different neurotransmitters. In the presence of catecholamines as reducing mediators, silver coats the surface of nanorods and leads to a multicolor shift in the absorbance spectra, collected as respond profiles. Different concentrations of GNRs and silver nitrate produce various thicknesses of silver nanoshells on the surface of GNRs and consequently generate different respond signals (see Figures S1 and S2 of the supporting information (SI)). The formation of the silver nanoshell is attributed to the reduction of silver ions by DA, EP and NE with different chemical structures (shown in Figure S3) and reduction strengths. Figure 1 demonstrates the response patterns collected for the discrimination of neurotransmitters at the concentration of 20 µg mL^−1^. The rest of the bar plots for the remaining concentrations (1–30 µg mL^−1^) can be seen in Figure S4. Thus, as shown in Table S1 the sensor array consisted of four cross-responsive sensor elements including two different concentrations of silver nitrate versus two concentrations of GNRs. In addition, the pH of the solution showed to affect the sensor responses. As shown in Figure S5, increasing pH resulted in the oxidation of catecholamines, while the silver atom deposition did not occur perfectly upon decreasing the pH. Therefore, pH 7 was chosen as the optimal value throughout the whole procedure. It has also been investigated the effect of GNRs AR (i.e. 7.0 and 4.0) on sensor array response. It was observed that GNRs with higher AR, response to a wider range of neurotransmitter concentrations (see Figure S6). Therefore the GNRs with AR 7.0 were selected for further experiments. In optimize condition a distinct pattern of colorimetric responses was achieved for selective detection and discrimination among DA, EP and NE (Figure S7).

### Sensor array responses

**Figure 2:**
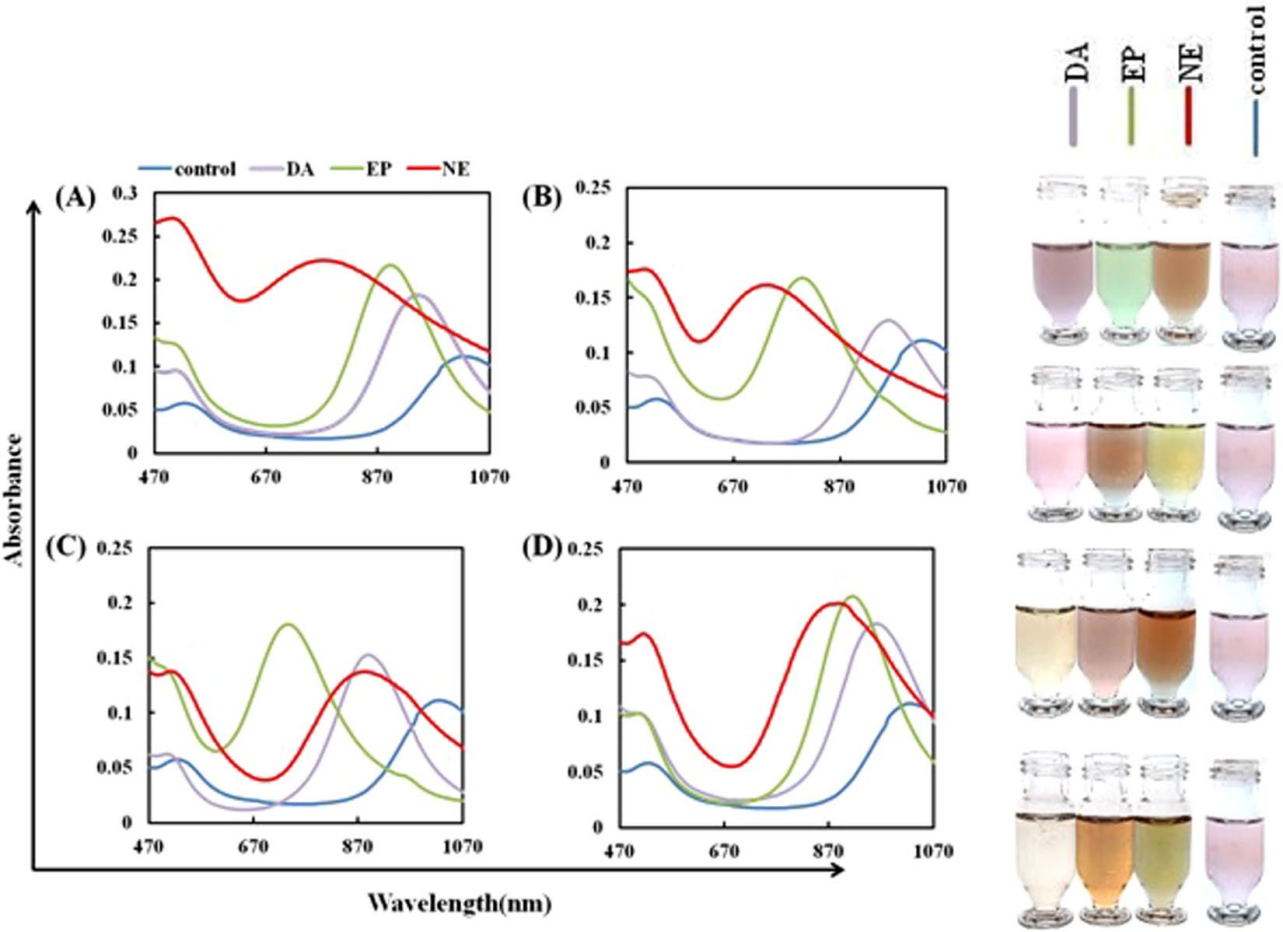
UV-visible spectra and corresponding images of (**A**) SE1, (**B**) SE2, (**C**) SE3 and (**D**) SE4: before and after addition of dopamine, epinephrine and norepinephrine (catecholamine at concentration of 20 µg mL^−1^).

Different concentrations of catecholamine neurotransmitters including DA, EP and NE ranging from 1 to 30 µg mL^−1^ were exposed to four sensor elements and spectral responses in 470 to 1100 nm were recorded (Figure S8). As illustrated in Fig. 2, the colors of the solutions changed from pink to orange and to green upon the addition of different neurotransmitters to the solution. No spectral nor color changes were observed in the absence of neurotransmitters (blank solution). The variations in the absorbance spectra were mainly related to the changes in the aspect ratio of GNRs which happened due to silver atom deposition on their surface. TEM and EDS analysis confirmed the deposition of nanoshell on the surface of GNRs (Figure S9A–D). As shown in Figure S9A and D, compared to A and C, the morphology of the GNRs has clearly changed; verifying the aspect ratio changes in the GNRs and the strong EDS peaks of silver deposited on the surface of GNRs. GNRs of 70 ± 5 nm in length and 10 ± 2 nm in diameter exhibit two peaks in their absorbance spectrum at about 1005 ± 5 nm and 520 nm, corresponding to their longitudinal and transverse surface Plasmon resonance, respectively. As mentioned earlier, the reduction of silver ions results in the formation of silver nanoshell on the surface of GNRs. Consequently, the changes in the aspect ratio of GNRs together with the optical properties of the silver nanoshell results in a blue shift in the longitudinal SPR band. The color of the solution, depending on the type of neurotransmitters, changes from pink to green. Figure 2 demonstrates the sensor elements response to DA, EP and NE in 20 µg mL^−1^ as an example. The response profile of each neurotransmitter is different from the others. The spectral shift and absorption intensity of each neurotransmitter changes upon the treatment of different concentrations of GNRs and silver nitrate. The spectral changes of GNRs after the reduction of silver ions were collected as response profiles of the sensor array.

### Statistical analysis

**Figure 3:**
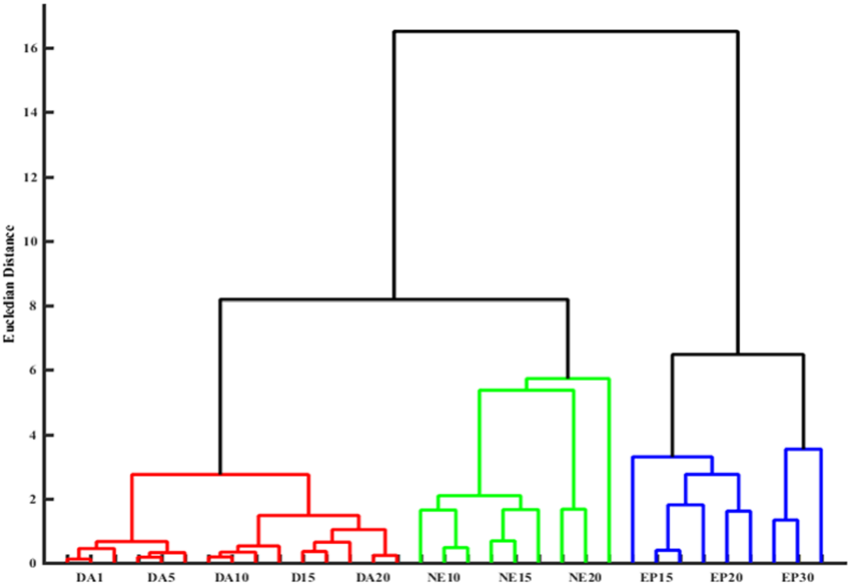
HCA dendrogram for catecholamine neurotransmitters. All the experiments were performed in triplicate. The concentration range of catecholamine neurotransmitters was 1–30 µg mL^−1^.

**Figure 4:**
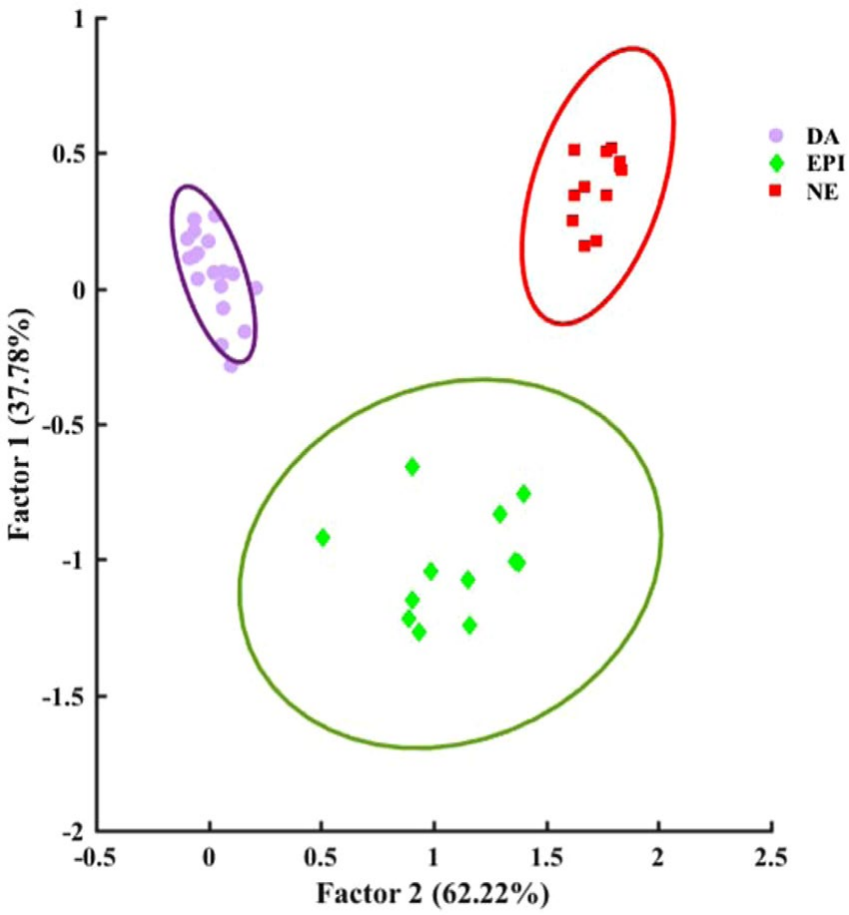
LDA score plot demonstrating clear differentiation between dopamine (1–30 µg mL^−1^), epinephrine (10–30 µg mL^−1^) and norepinephrine (10–30 µg mL^−1^).

The collected responses were processed using standard chemometric methods. Principal component analysis (PCA), hierarchical cluster analysis (HCA) and linear discriminant analysis (LDA) were used to assess the potential of the colorimetric sensor array for simultaneous detection of catecholamine neurotransmitters. In order to quantitatively classify the observed spectral changes of the array, four wavelengths of the response spectra were selected based on more variations corresponding to each sensing element (i.e., 520, 850, 950, and 1000 nm). Based on the ∆A values (i.e., difference between the absorbance after analyte addition and the blank) 16-dimensional vectors consisting of four sensor elements and four wavelengths were defined for each catecholamine. In other words, each neurotransmitter had a distinct pattern which was used for its recognition. HCA as an agglomerative clustering method^24^ was performed using Ward’s minimum variance for different neurotransmitters at different concentrations. As shown in HCA dendrogram (Fig. 3), dopamine in concentration range of 1–20 µg mL^−1^, epinephrine in 20–30 µg mL^−1^ and norepinephrine in range of 10–20 µg mL^−1^ were classified without misclassification in triplicate trials. Figure S10 demonstrates well classified score plots of catecholamine neurotransmitters obtained by the first two principal components (PC1, PC2). PC1 captured 75.6% of total variance while PC2 captured 13.29%; therefore, approximately 90% of the variance was described by these two PC axes. Thereafter, LDA was used which is normally employed in statistics to find a linear combination of features that can differentiate two or more classes of objects or events^51^. LDA analysis reduced the size of the training matrix (4 sensing element × 3 catecholamine neurotransmitter × 3 replicates) and transformed them into canonical factors. Therefore, two-dimensional score plots with classification accuracies of 100% were achieved (see Fig. 4). Based on the LDA results, two canonical factors (62.22% and 37.78%) displayed 100% of the variance in the data, and the neurotransmitters were clearly classified into 3 distinct groups by this pattern recognition method. The results also demonstrate that these similar analytes were perfectly differentiated in various concentrations.

### Color difference map of the array

**Figure 5:**
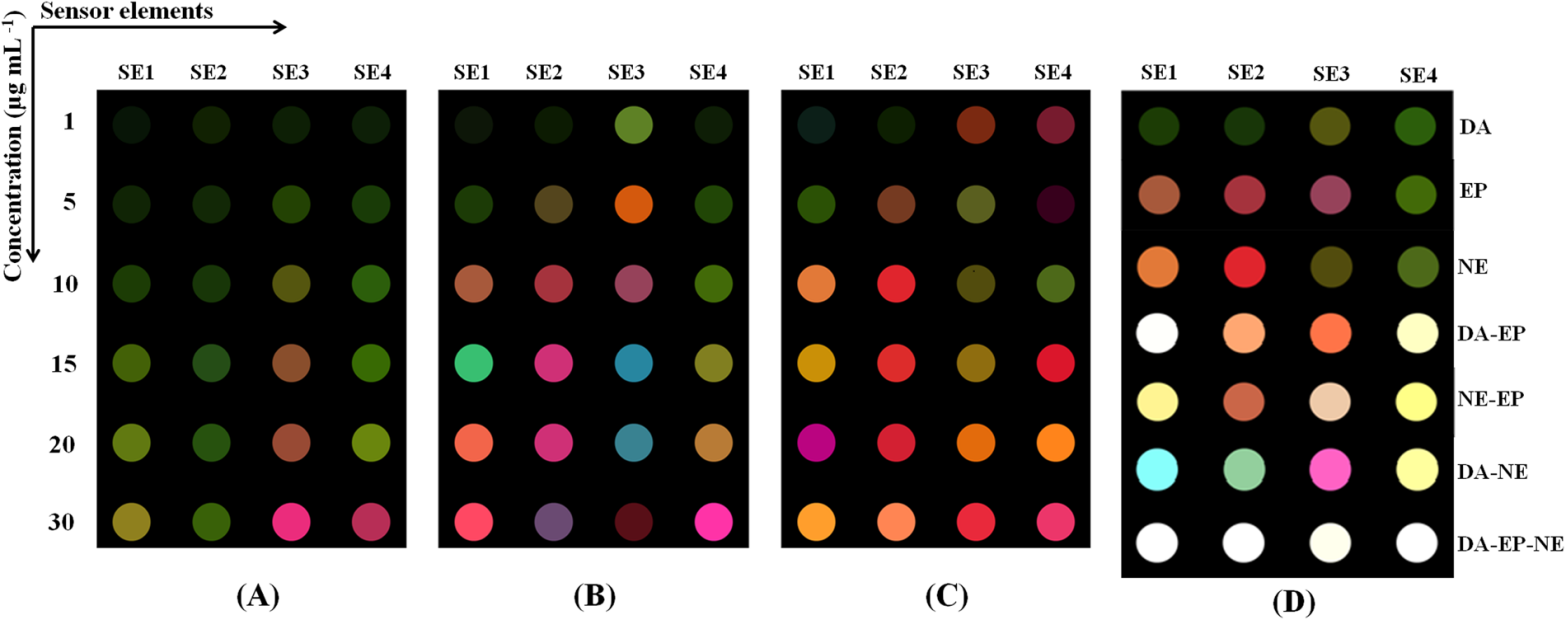
Unique color patterns as fingerprints for (**A**) DA, (**B**) EP and (**C**) NE at different concentrations (1–30 µg mL^−1^). (**D**) Sensor array responses for binary and ternary mixtures of the neurotransmitters at concentration of 10 µg mL^−1^.

Color difference maps, as useful qualitative tools for visualization of the colorimetric sensor array responses, were depicted by subtracting of absorbance before and after exposure to catecholamine neurotransmitters at three visible wavelengths (i.e., 850, 950 and 1000 nm). Color difference maps presented in Fig. 5; demonstrate unique color patterns as fingerprints for each analyte in different concentrations (1–30 µg mL^−1^). These fingerprints allow simultaneous detection of neurotransmitters even without need to statistical techniques. The limit of detection (LOD) and limit of recognition (LOR) based on color difference maps^52^ are shown in Table S2.

### Correlation between total Euclidean distance (T.E.D.) and catecholamine concentration

The relationship between the T.E.D. of ∆A sensor elements (i.e., square root of sums of squares ∆A values) was probed as a function of catecholamine concentration (Figure S11). Increasing the concentration of neurotransmitters is associated with gradual increase in T.E.D. A linear response was observed for dopamine in the range of 1–30 µg mL^−1^ and respectively for epinephrine and norepinephrine in the ranges of 10–30 µg mL^−1^ and 10–20 µg mL^−1^. Based on the results, proposed colorimetric sensor array can be used for qualitative and semi quantitative identification of the catecholamine neurotransmitters using LDA, HCA and color difference map.

### Mixture analysis

One of the great advantages of array-based sensors is their ability to simultaneously determine similar looking analytes in complex mixtures. Without the array strategy, it might not be possible to determine analytes with similar chemical structures coexisting in a solution. The reason might be attributed to the similar response rising from similar analytes, preventing individual determinations without interfernces. Thus, the implementation of array-based strategies provides a wider response space and the high dimensionality of the responses actually allows simultaneous detection of these analytes. In this study, the ability to simultaneously determine DA, EP and NE would be very useful in clinical diagnosis. For instance, they may be applied in understanding the disease mechanisms especially in tumor cells’ malignancy, early cancer screening and follow up after anticancer drug treatment^53^.

So, simultaneous detection of DA, EP and NE is far more challenging from their individual detection because of their similar structure. In this regards, the sensor array responses for binary and ternary mixtures of the neurotransmitters at concentration levels of 10 µg mL^−1^ were recorded. It was found that binary (DA-EP, DA-NE and EP-NE) and also ternary mixtures (DA-EP-NE) showed different responses, compared to individual catecholamines. LDA and PCA score plots for 10 µg mL^−1^ of each neurotransmitter (both individually and in mixture) demonstrate that all mixtures were separated by 100% cross-validation accuracy (Figures S12 and S13). Moreover, as shown in Fig. 5D, color difference map of mixtures visually shows completely different response patterns from the individual neurotransmitters.

### Detection of neurotransmitters in real samples

**Figure 6:**
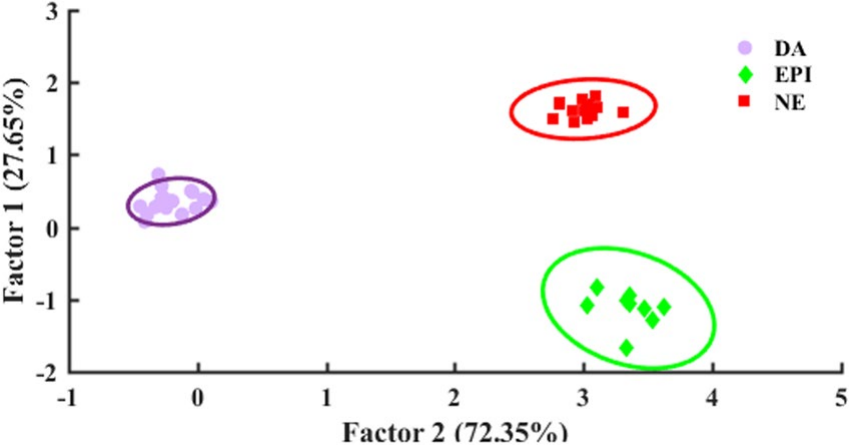
LDA score plot, urine sample was spiked with catecholamines at concentrations of 10 µmol L^−1^.

Finally, the potential applicability of the sensor array for catecholamine neurotransmitters detection in human urine sample was evaluated. As shown in Fig. 6, the array was able to discriminate DA, EP and NE which reveals the high potential of the designed sensor array for their simultaneous detection.

## Conclusions

In summary, two different concentrations of GNRs and two concentrations of silver nitrate were employed as simple sensing elements to design a colorimetric sensor array for the detection and discrimination of catecholamine neurotransmitters, including dopamine, epinephrine and norepinephrine. The difference in silver reducing ability of catecholamines led to the formation of different thicknesses of silver nanoshell on the surface of GNRs, which resulted in different LSPR longitudinal peak shifts. Though this sensing mechanism has been previously applied for the detection of various individual analytes, this is the first time that this idea is successfully being used in a sensor array design in order to differentiate among different neurotransmitter. Furthermore this system is able to discriminate catecholamine neurotransmitters in complex mixtures, as well as urine samples.

## Methods

### Reagents

Hydrogen tetrachloroaurate (HAuCl_4_.3H_2_O), silver nitrate (AgNO_3_), sodium borohydride (NaBH_4_), cetyltrimethylammonium bromide (CTAB), 5-bromosalicylic acid (5-Br-SA), ascorbic acid (AA), sodium hydroxide (NaOH), hydrochloric acid (HCl), and dopamine hydrochloride (DA), epinephrine hydrochloride and nor epinephrine hydrochloride were obtained from Sigma. All the chemicals used were of analytical reagent grade and Milli-Q water was used throughout.

### Instrumentation

Acquisition of absorbance spectra was performed with a Lambda 25 spectrophotometer from Perkin Elmer and the use of 1.0 cm glass cells. These spectra were recorded at room temperature. Measurements of pH were performed with a Denver Instrument Model of 270 pH meter equipped with a Metrohm glass electrode. Transmission electron microscopy (TEM) images were recorded with a PHILIPS MC 10 TH microscope (USA) at an accelerating voltage of 100 kV. Energy-dispersive spectroscopy (EDS) measurements were carried out on a Sirius SD (UK) energy dispersive spectrometer.

### Preparation of GNRs

A stock solution of GNRs was prepared according to the method reported by Ye *et al*.^54^ with slight modification. Briefly, the seed solution was prepared by the addition of 5.0 mL of 0.1 mol L^−1^ of CTAB to 0.05 mL solution of 2.5 × 10^−2^mol L^−1^ HAuCl_4_. Afterward, 0.3 mL of 0.01 mol L^−1^ ice-cold NaBH_4_ was injected to this solution under vigorous stirring. The resulting brownish yellow solution was stirred for 3 min and left at room temperature for about 2 h. For preparing the growth solution, 0.015 g 5-Br-SA was added to 50 mL of 0.05 mol L^−1^ of CTAB under moderate stirring, followed by the addition of 1.15 mL of 0.01 mol L^−1^ AgNO_3_. The mixture was kept untouched for 15 min, then 1 mL of 2.5 × 10^−2^mol L^−1^ HAuCl_4_, 0.40 mL of HCl (37 wt% in water, 12.1 mol L^−1^) and 0.30 mL of 0.10 ascorbic acid were added respectively. Eventually, 0.06 mL of CTAB-capped gold seeds was added into the solution under gentle stirring. The color of the solution gradually changed within 1 h, and the solution left undisturbed overnight at 27 °C to complete the growth process. Concentration of GNRs was estimated to be 0.05nmol L^−1^ according to a previously published method^55^. The GNRs were collected, centrifuged twice (10000 rpm, 10 min), and dispersed in Milli-Q water. The excess CTAB was removed by centrifugation and the pH of final solutions was adjusted to 7.0.

### Colorimetric sensor array design and neurotransmitters detection

Two concentrations of GNRs (i.e., 0.001 and 0.015nmol L^−1^) and two concentrations of silver nitrate (i.e., 0.3 and 0.5 mmol L^−1^) were selected as sensing elements (see Table S1). For instance, the first sensor element (SE1) consisted of 0.001nmol L^−1^ of GNRs and 0.3 mmol L^−1^ of silver nitrate. For the detection step, different amounts of analytes were added to each of the four sensing solutions. UV-Vis spectra were recorded at room temperature after 20 min of adding last drop of analytes.

### Preparation of urine sample

Urine was obtained from a healthy volunteer. The urine samples were diluted 100 times with Milli-Q water and were then spiked with catecholamines.

## Electronic supplementary material

SUPPLEMENTARY INFO
