# Supplementary material for: Colorimetric Fingerprints of Gold Nanorods for Discriminating Catecholamine Neurotransmitters in Urine Samples
Source: Sci Rep. 2017 Aug 15;7:8266. doi: 10.1038/s41598-017-08704-5 (PMC5557886; doi:10.1038/s41598-017-08704-5)
Supplement: Supplementary file 1 — SUPPLEMENTARY INFO [file 41598_2017_8704_MOESM1_ESM.doc]

**Colorimetric Fingerprints of Gold Nanorods for Discriminating Catecholamine Neurotransmitters in Urine Samples**

**Somayeh Jafarinejad^1^, Mahmoud Ghazi-Khansari^2^, Forough Ghasemi^3^, Pezhman Sasanpour^1^* & M. Reza Hormozi-Nezhad^3^***

^1^Department of Medical Physics and Biomedical Engineering, Faculty of Medicine, Shahid Beheshti University of Medical Sciences, Tehran, Iran

^2^Department of Pharmacology, School of Medicine, Tehran University of Medical Sciences, P.O. Box 13145-784, Tehran, Iran

^3^Department of Chemistry, Sharif University of Technology, Tehran, 11155-9516, Iran

*Corresponding authors: (M.R.H) email: [hormozi@sharif.edu](mailto:hormozi@sharif.edu); (P.S) email: [pesasanpour@sbmu.ac.ir](mailto:pesasanpour@sbmu.ac.ir)

**Table S1.** Illustration of the designed 2×2 array of gold nanorods-silver nitrate.

| C_Ag+_  C_AuNRs_ | 0.5mmol^-1^ | 0.3mmol L^-1^ |
| --- | --- | --- |
| 0.001nmol L^-1^ | SE_1_ | SE_2_ |
| 0.015nmol L^-1^ | SE_3_ | SE_4_ |

**Table S2:** Figures of merits for catecholamine neurotransmitters based on T.E.D. and color difference map.

|  | Dopamine | Epinephrine | Norepinephrine |
| --- | --- | --- | --- |
| Linear Range (µg mL^-1^) | 1-30 | 10-30 | 10-20 |
| LOD (µg mL^-1^) | 5.0 | 1.0 | 1.0 |
| LOR (µg mL^-1^) | 1.0 | 1.0 | 1.0 |


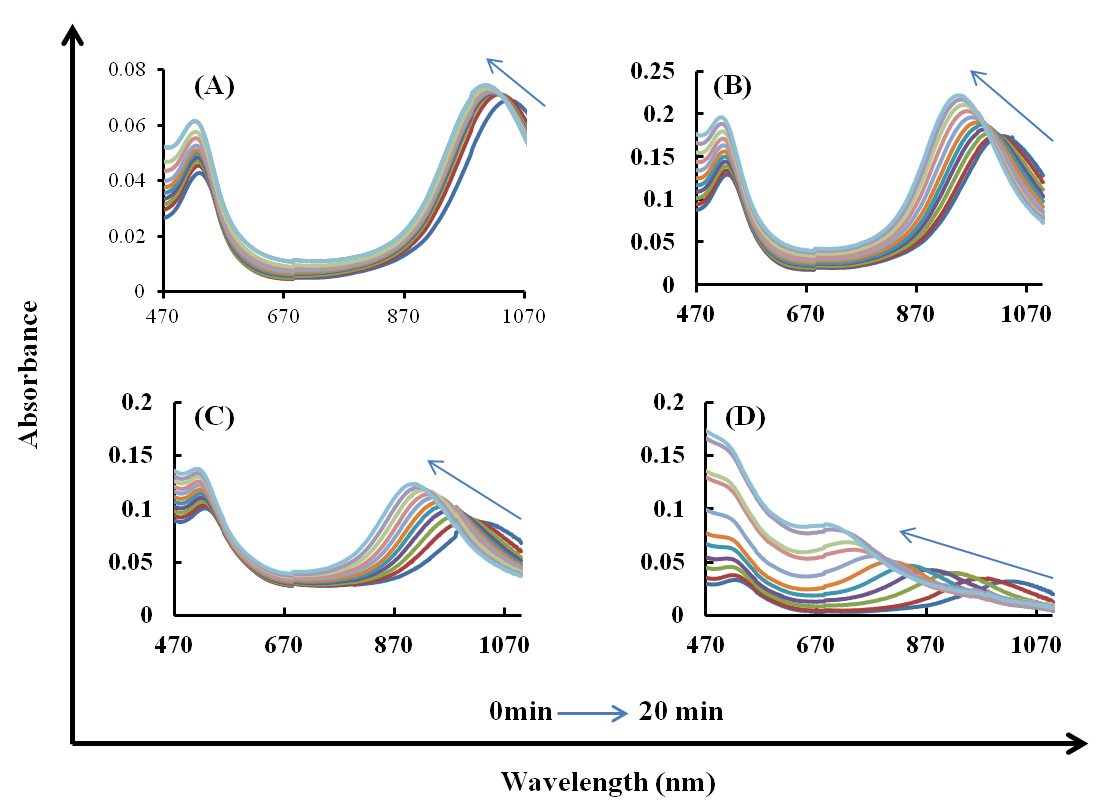


**Figure S1.** Effect of GNRs concentration ((A):0.005nmol L^-1^ (B): 0.0025nmol L^-1^(C):0.001nmol L^-1^ and (D):0.0005nmol L^-1^) on their LSPR longitudinal peak in the presence of same concentration of dopamine (10µg mL^-1^) and silver nitrate (0.25mmol L^-1^ at 2min intervals until 20min).


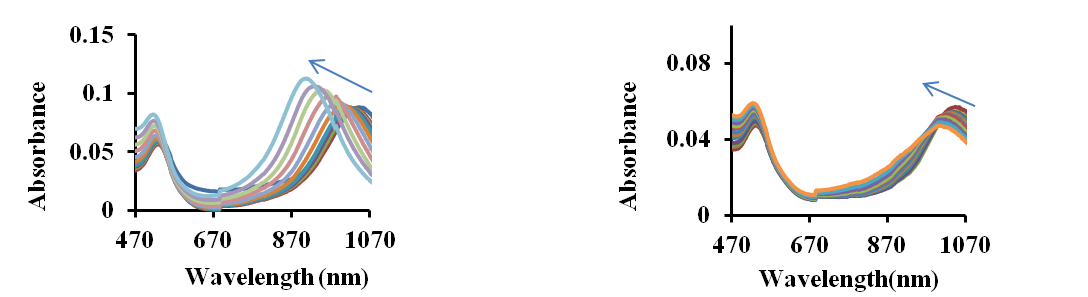


0 min → 20 min

**Figure S2.** Effect of silver nitrate concentration (A): 0.25mmol L^-1^ (B): 0.15mmol L^-1^ on the GNRs longitudinal peak shift in presence of same concentration of dopamine (10µg ml^-1^) and GNRs (0.001nmol L^-1^) during 20min.

**
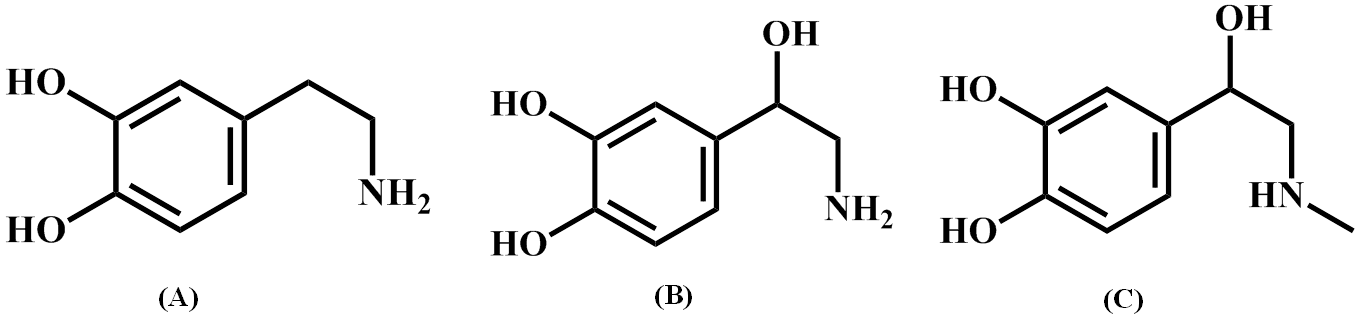
**

**Figure S3.** Chemical structure of catecholamine neurotransmitters (a) Dopamine, (b) Epinephrine, and (c) Norepinephrine.


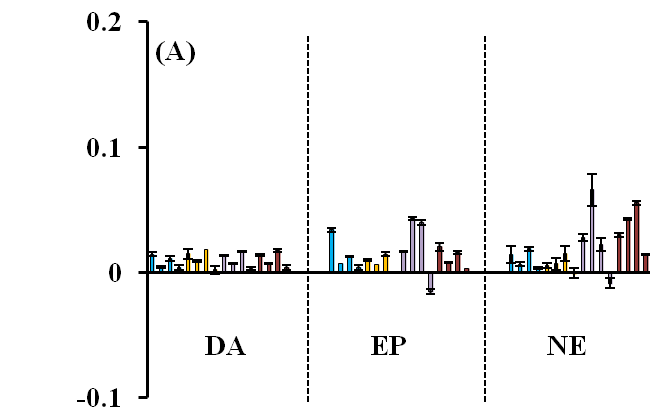

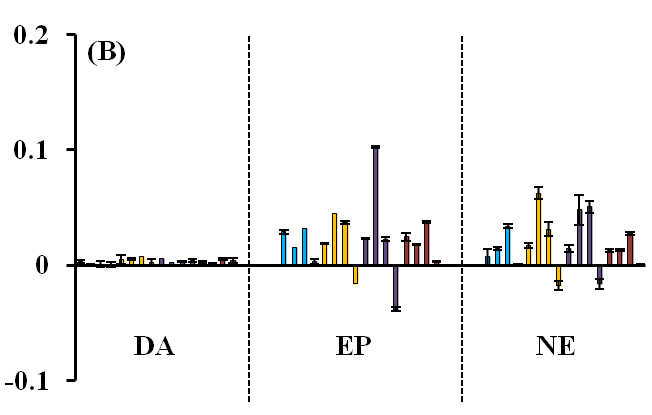


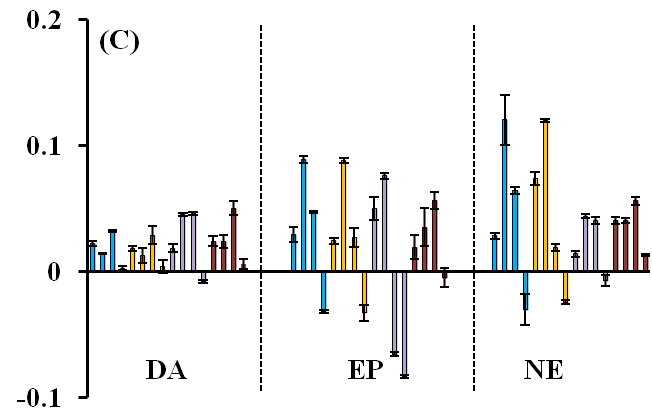


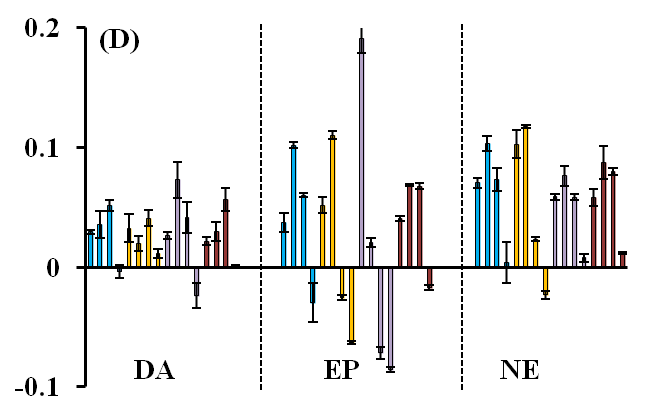


**A-A_0_**

**
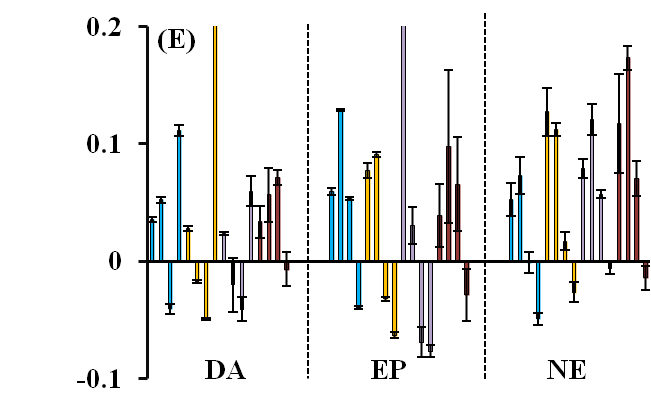

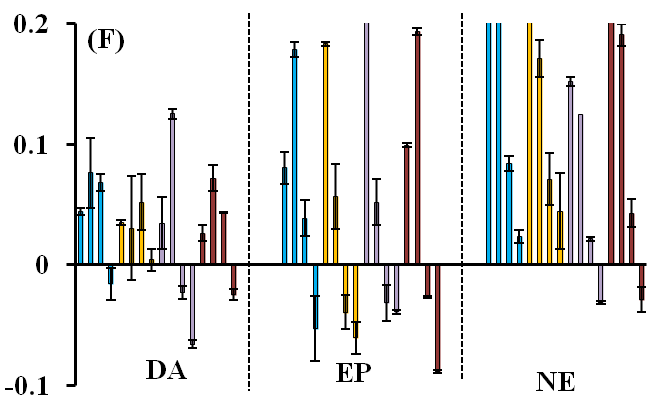
**

**Catecholamine Neurotransmitters**

**Figure S4.** Responses of various sensor elements (SE1, SE2, SE3 and SE4) to different catecholamine neurotransmitters in concentration of 1µg mL^-1^ (A), 5µg mL ^-1^ (B), 10µg mL^-1^ (C), 15µg mL^-1^ (D), 20µg mL^-1^ (E) and 30µg mL^-1^ (F).


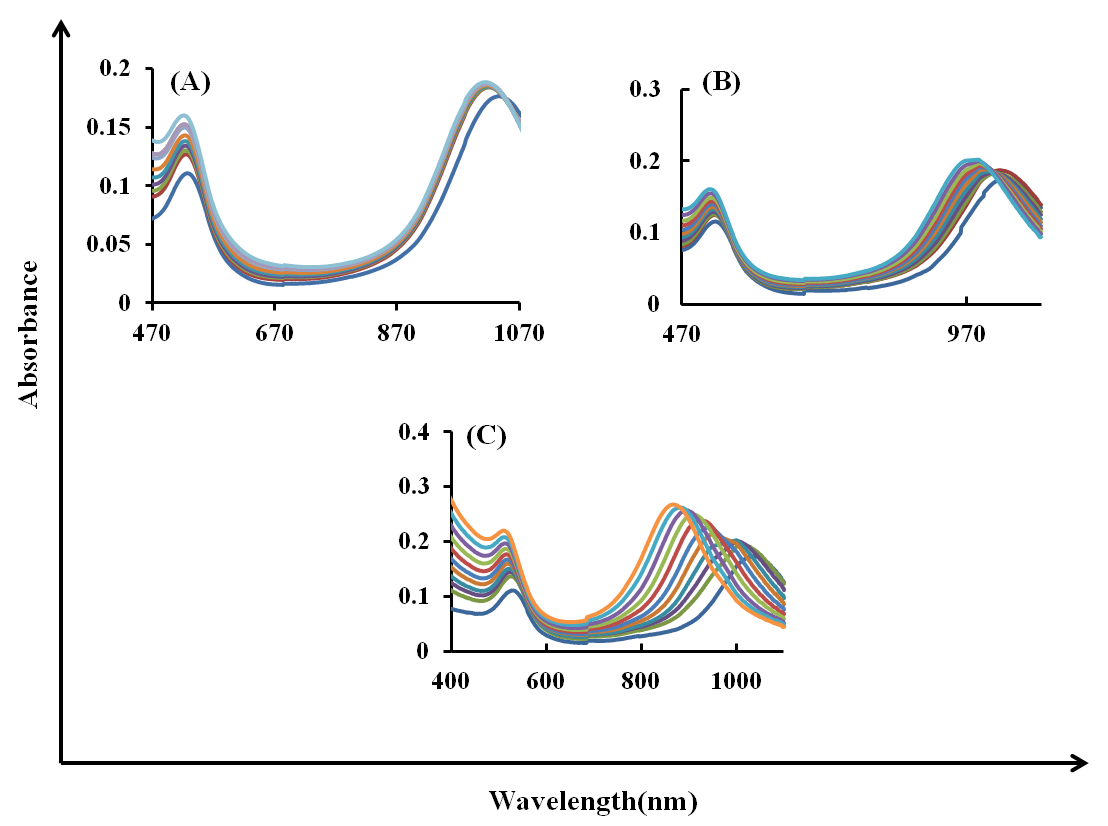


**Figure S5.** The effect of pH in spectral responses of GNRs during 20min. A) pH:5.0, B) pH:6.0 and C) pH:7.0.


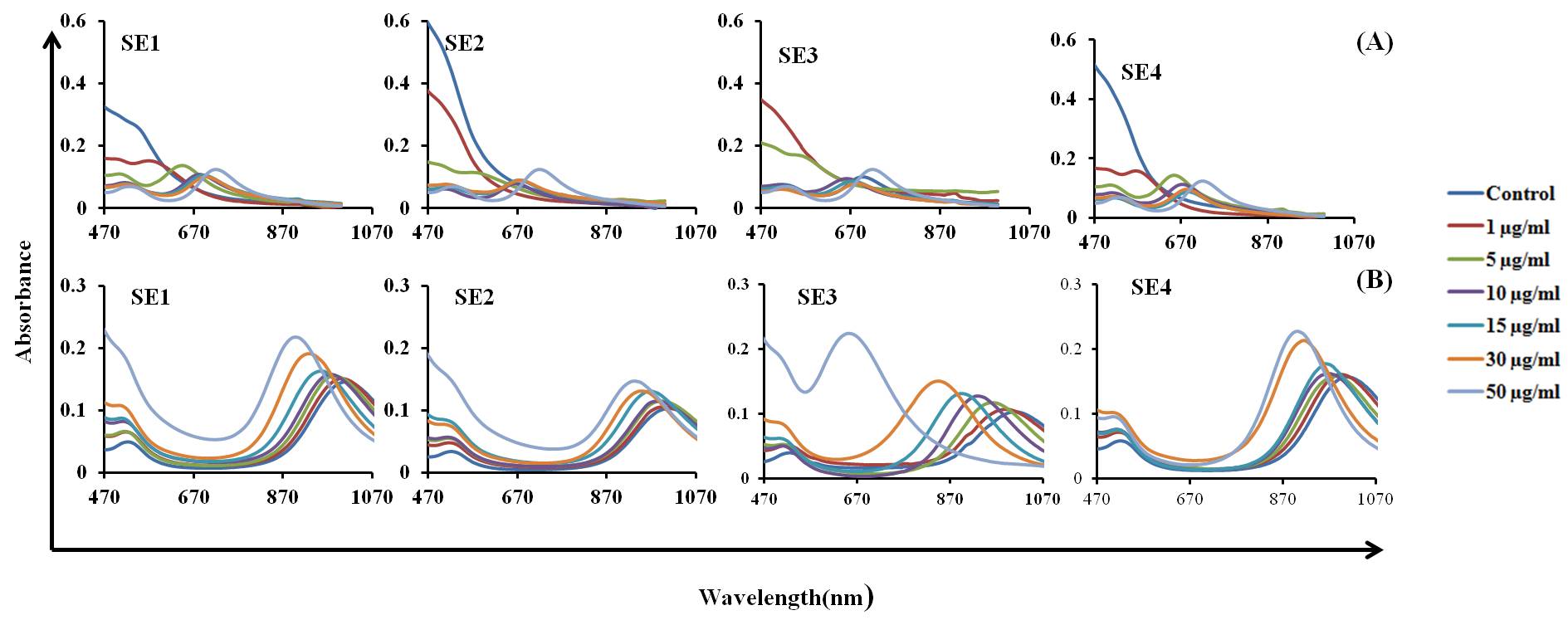

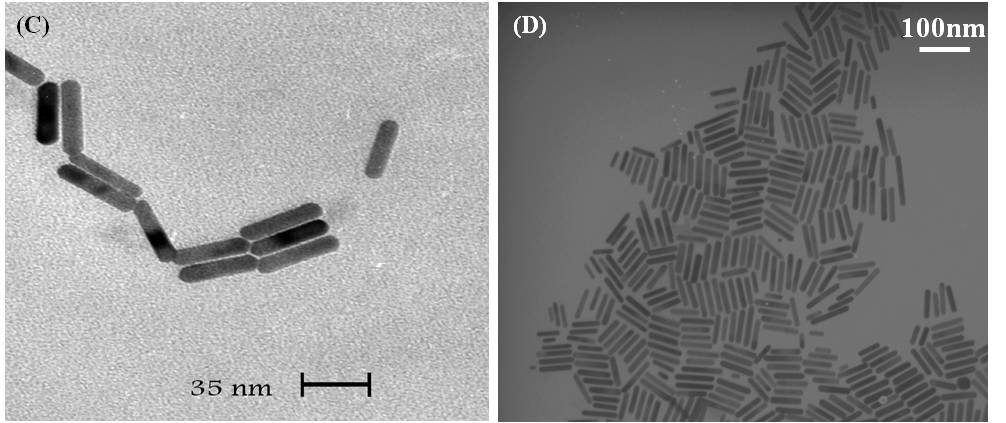


Figure S6: The effect of GNRs aspect ratio (A) 4.0 and (B) 7.0 on sensor elements response. TEM image of GNRs with aspect ratio (C) 4.0 and (D) 7.0.


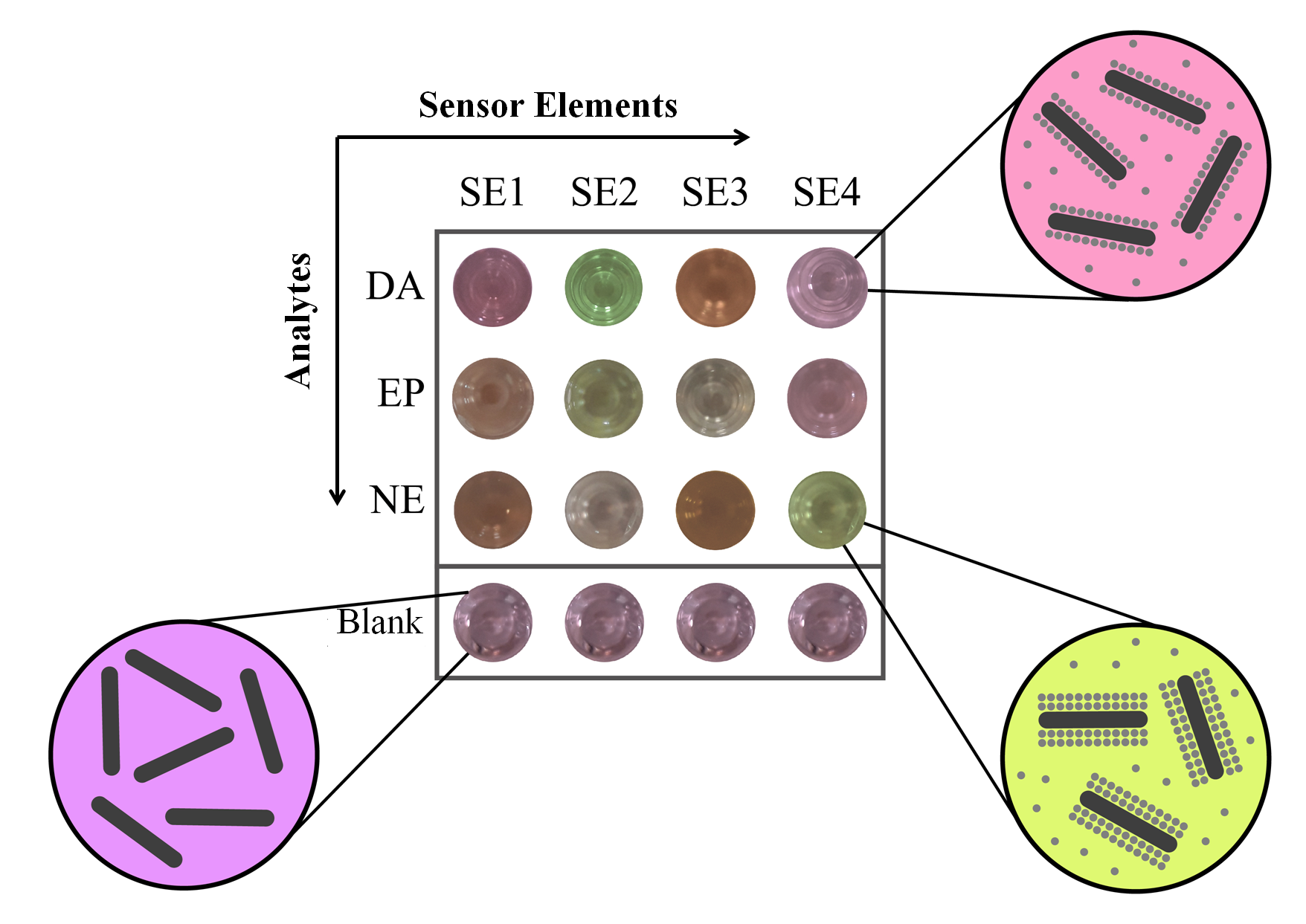


**Figure S7.** Distinct pattern of colorimetric sensor array and detection principle of catecholamine neurotransmitters based on silver atom deposition on the surface of GNRs.


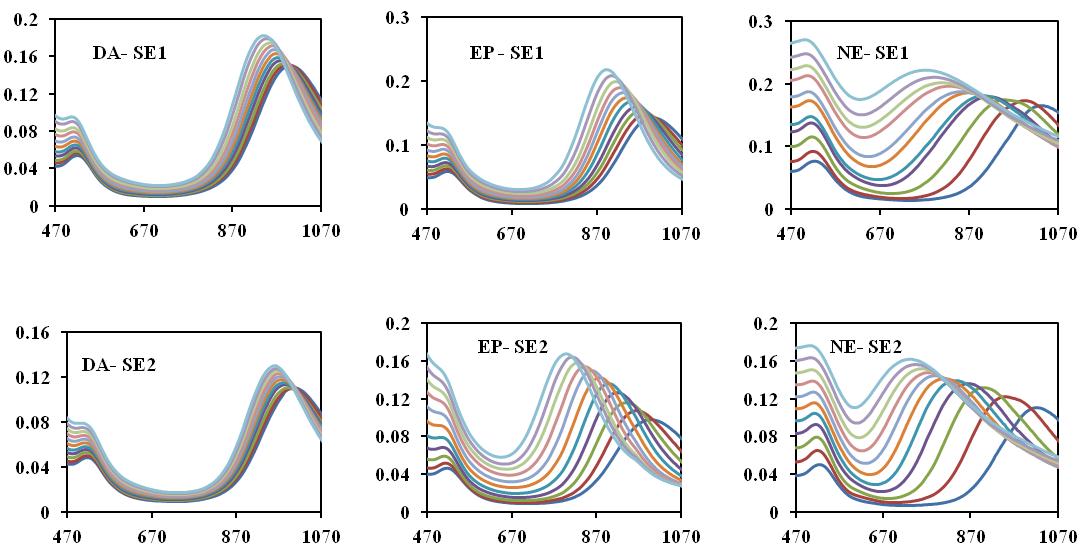


**20min 0 min**


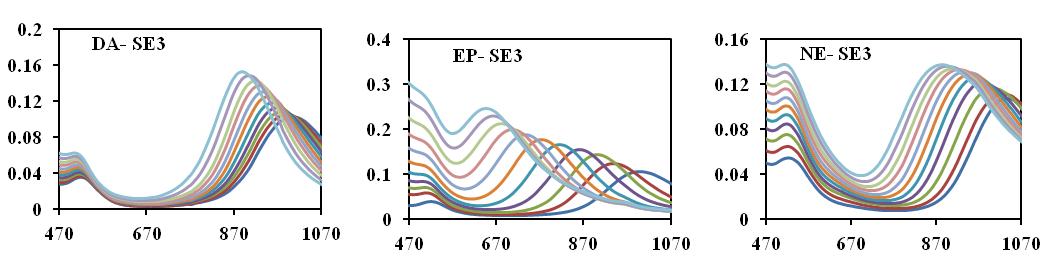

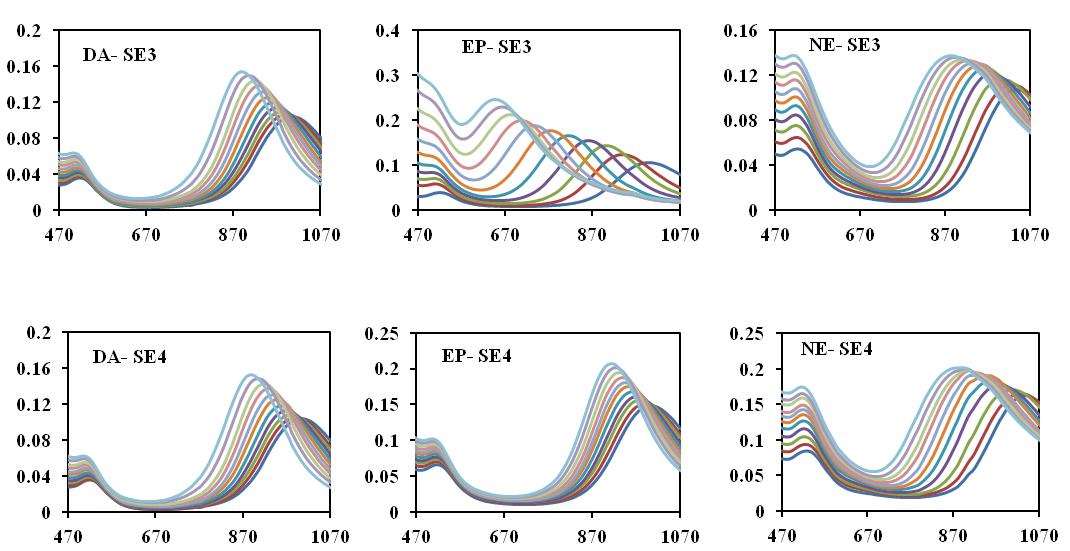


**Absorbance**

**Wavelength (nm)**

**Figure S8.** Spectral responses of dopamine (DA), epinephrine (EP) and norepinephrine (NE) versus four sensor elements (SE1-SE4) in concentration of 20µg mL^-1^ at 470nm to 1100nm wavelength in every 2min till 20min.


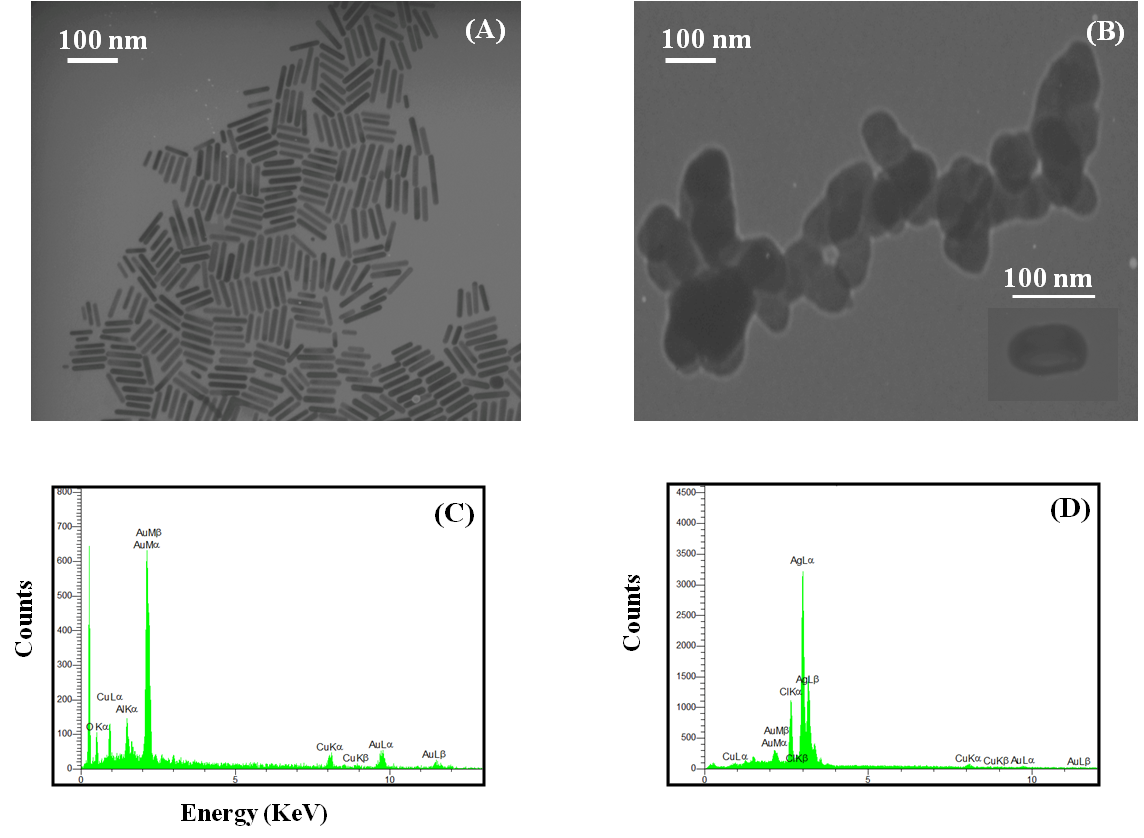


**Figure S9.** TEM images and EDS analysis of GNRs before reaction (A, C) and after reaction (B, D).


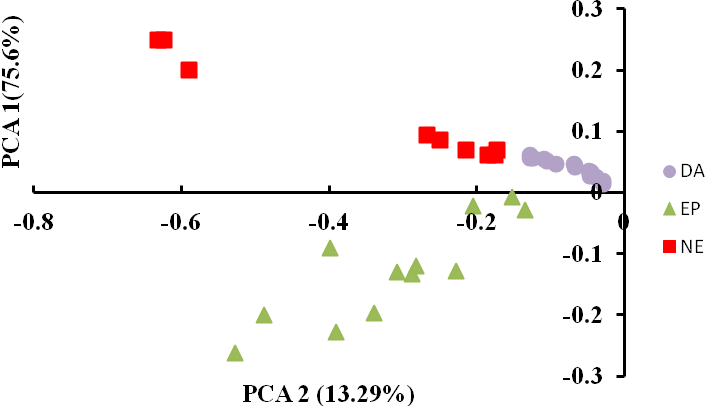


**Figure S10.** PCA score plot for determination and discrimination of dopamine(DA), epinephrine(EP) and norepinephrine(NE).


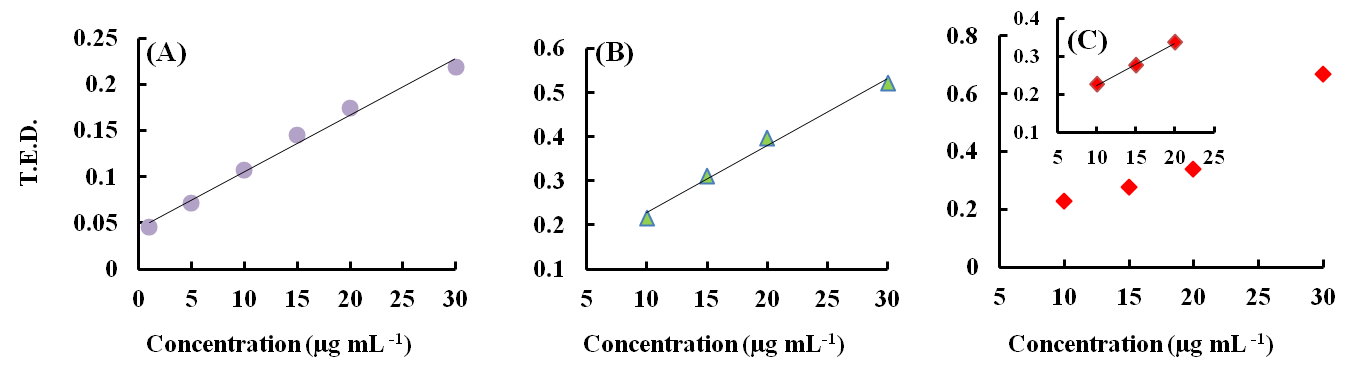


**Figure S11.** The relationship between the T.E.D. of ∆A sensor as a function of catecholamine concentration. (A) Dopamine, (B) Epinephrine and (C) Norepinephrine.


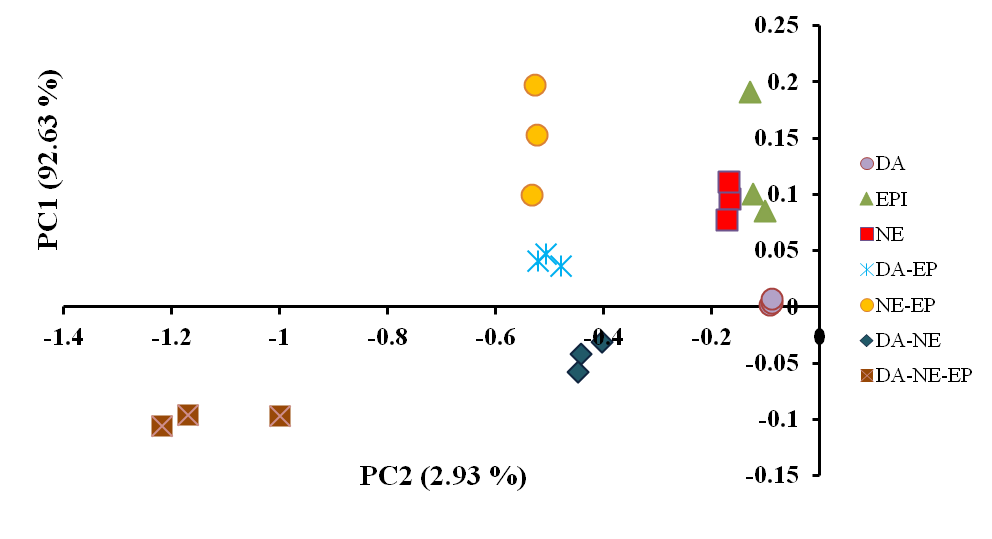


**Figure S12.** PCA score plots for 10µg mL^-1^ of each neurotransmitter in mix solution.


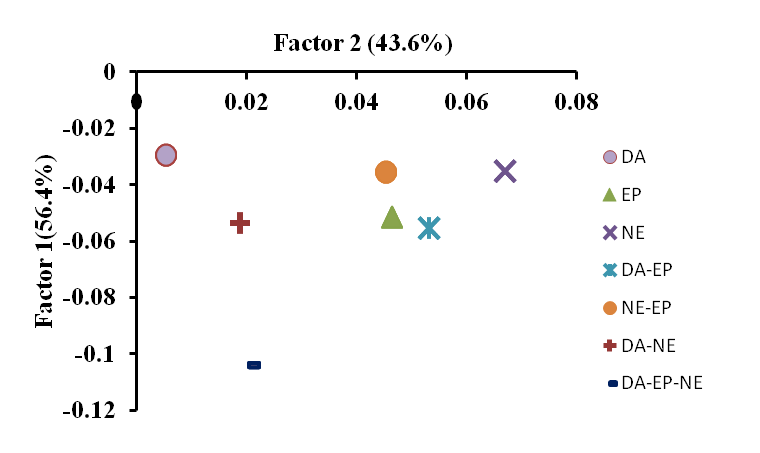


**Figure S13.** LDA score plots for 10µg mL^-1^ of each neurotransmitter in mix solution.
